# Cavernous hemangioma of the parotid gland in adults

**DOI:** 10.4317/jced.51750

**Published:** 2014-12-01

**Authors:** Hugo Lara-Sánchez, Beatriz Peral-Cagigal, Beatriz Madrigal-Rubiales, Alberto Verrier-Hernández

**Affiliations:** 1 M.D, ENT Resident. Otolaryngology Head and Neck Surgery Department. Río Hortega University Hospital.Valladolid, Spain; 2 M.D, Oral and Maxillofacial Surgeon. Oral and Maxillofacial Surgery Department. Río Hortega University Hospital. Valladolid, Spain; 3 M.D, Pathology Physician. Pathology Department. Río Hortega University Hospital. Valladolid, Spain; 4 Chief of Oral and Maxillofacial Surgery. Oral and Maxillofacial Surgery Department. Río Hortega University Hospital. Valladolid, Spain

## Abstract

Hemangiomas account for 0.4-0.6% of all tumors of the parotid gland and most of them occur in children, nevertheless in adults hemangiomas are very rare. We report the case of a 62 year old woman with a mass in the parotid right tail associated with fluctuating swelling episodes unrelated to meals and with a slowly progressive growth. The provisional diagnosis was a pleomorphic adenoma, so a right superficial parotidectomy was performed. During surgery, the macroscopic appearance makes suspect a vascular lesion. The histopathological result was a cavernous hemangioma. The classic clinical presentation of a parotid hemangioma is an intraglandular mass associated or not with skin lesions characterized by reddish macules and/or papules, and a vibration or pulsation when palpating the parotid region. In imaging tests, phleboliths could be observed which are very suggestive of a hemangioma or a vascular malformation. In the absence of these signs, the diagnosis could be difficult, particularly in an adult due to its low prevalence, with about 50 cases reported worldwide. However a hemangioma should be considered in the differential diagnosis of parotid tumors in adults.

** Key words:**Cavernous hemangioma, parotid gland, superficial parotidectomy, pleomorphic adenoma.

## Case Report

Hemangiomas are vascular abnormalities that are characterized by increased proliferation and renewal of endothelial cells. They are classified as cavernous, capillary and mixed hemangiomas (1). The 65% of the hemangiomas are located in the head and neck and they principally affect the salivary glands with the parotid as the most common site (81-85 %). Hemangiomas account for 0.4-0.6% of all tumors of the parotid gland and most of them occur in children, nevertheless in adults hemangiomas are very rare (2,3).

A 62 years old female with no family history of interest has a right parotid mass with a slowly progressive growth of 4 years of evolution. Initially, it was asymptomatic and subsequently, fluctuating swelling episodes, not associated with meals, in the parotid region appeared.

**Figure 1:**
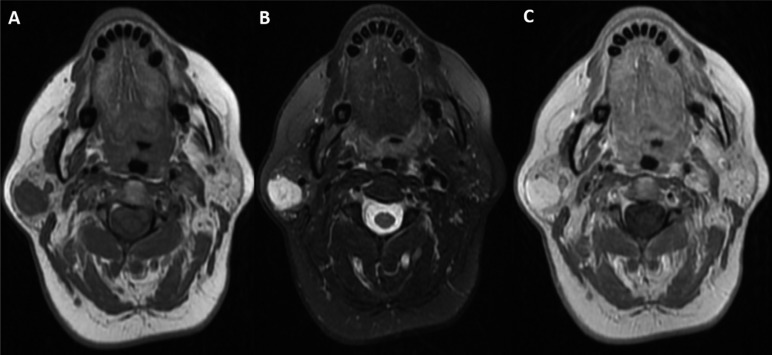
A) MRI T1 reveals in the right superficial lobe of the parotid gland a well defined, hipointense and lobulated intraparotid tumor of approximately 3cm. It is located posterior and lateral to the retromolar vein. Rest of the glandular parenchyma has normal morphologic features and intensity. B) MRI T2, shows the same image but the tumor is hyperintense. C) MRI T1 with contrast, shows the same image but with intense enhancement of the tumor.

On physical examination, a soft, elastic, painless, non-fluctuating, non-pulsatile mass without trophic skin changes and of 3x2cm of maximum diameter, was palpated in the tail of the right parotid gland. The parotid duct was permeable with transparent production of saliva. Cervical lymphadenopathy or other masses were not palpable. Fine needle aspiration (FNA) and magnetic resonance imaging (MRI) was made to guide the diagnosis. The result of FNA was inconclusive. In the MRI, at the superficial lobe of the parotid gland, posterolateral to the retromandibular vein, a well-defined mass 3 x 2.6 x 2.3 cm was revealed. It appeared as a lobulated tumor, hyperintense on T2, hypointense on T1, and with intense and homogeneous enhancement after intravenous contrast administration but without extension to adjacent structures (Fig. 1).

The provisional diagnosis is a clinical and radiological benign parotid tumor: a pleomorphic adenoma as the suspected diagnosis. So it was decided to perform a right superficial parotidectomy. During surgery, the macroscopic appearance was of a not encapsulated and hemorrhagic, well-defined tumor that suggests a vascular lesion.

**Figure 2:**
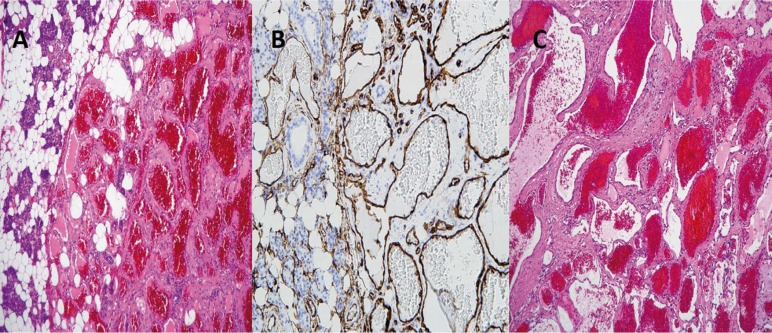
A) Microscopic image that reveal a vascular proliferation of different caliber, mostly wide dilated and congestive, that shows a lobular distribution pattern. The vessels are covered with flattened endothelium without atypia and with a thin wall supported by a dense collagen layer. In the periphery of the proliferation, parotid gland parenchyma is shown with predominance of serous acini and chronic inflammatory infiltration (H&E 45X). B) Microscopic image with immunohistochemical technique, which is CD34+. The vascular endothelium of the tumor is enhanced (100X). C) Microscopic image that shows in greater detail the vascular proliferation (H&E 100X).

The histopathological study reports a tumor with vascular proliferation of different caliber, mostly of them widely dilated and congestive, that showed a lobar pattern distribution. The vessels are lined by flattened endothelium without atypia and with a thin wall supported by a dense collagen layer. Centrally a hyalinized stroma is identified (Fig. 2). All suggestive of a cavernous hemangioma of the parotid gland.

At a 6 months of follow-up, the patient was asymptomatic, with a favorable clinical course, and without the presence of tumor recurrence.

## Discussion

The classic clinical presentation of a parotid hemangioma is the presence of a mass at the parotid region associated or not with skin lesions characterized by red, red-bluish, or blue macules and/or papules, as well as a vibration or pulsation when palpating the parotid region. The presence of radiological phleboliths is very suggestive of hemangioma or vascular malformation, however these only occur in 2-3% of cases and it should be differentiated from sialoliths by a sialography. If this signs are absent as in this case report, the diagnosis could be challenging, particularly in an adult patient in whom this disease is not suspected as the main possibility (4,5).

There are multiple reports of capillary hemangiomas of the parotid gland in pediatric population, which generally tend to involve. Cavernous hemangiomas in adults does not regress, and they tend to have a chronic course and a slowly progressive growth (5).

Due to the low prevalence of hemangiomas in adults, with about 50 cases reported worldwide (6). They are not usually taken into consideration in the differential diagnosis of parotid masses. Therefore, recurrent mumps, tumors or cystic lesion of glandular origin or hypertrophy of the masseter muscle are the principal differential diagnosis. In adults, the pleomorphic adenoma and the Warthin tumor are within the most common benign tumors of salivary glands (2).

Magnetic resonance imaging (MRI) is useful in demonstrating lesions of the parotid region and its extension. Hemangiomas usua-lly appear as a lobulated lesion with intermediate signal on T1, hyperintense on T2 and homogeneous enhancement with contrast. MRI also helps determine the surgical approach for the tumor and to reveal the relationship with adjacent structures. The fine needle aspiration (FNA) is useful in the preoperative diagnosis of tumors of the head and neck. It is considered unnecessary in a hemangioma because of the probability of generating a hematoma and when MRI is highly suggestive of the diagnosis. Therefore, a typical clinical presentation and characteristic radiologic findings are sufficient for the diagnosis (7,8).

Currently, the treatment of choice in the cavernous intraparotid hemangioma is surgery, taking into consideration a pre-surgical embolization. However, infantile hemangiomas have other treatment options such as endovascular sclerotherapy, intralesional or systemic corticosteroids, vincristine, and propanolol (9).

Researching studies have found recently, the expression of the cyclo-oxygenase 2 (COX2) protein on endothelial cells of various vascular spaces of cavernous hemangiomas. There is little evidence of the relation of vascular tumors with the expression of COX2. However, there has been reported that high doses of celecoxib have inhibited the cell proliferation of angiosarcomas cell lines. So it could be considered as a new therapeutic line research for tumors of vascular origin (10).
